# The mechanical and optical properties of different transparent vinyl polysiloxane materials used for guided composite resin injection technique

**DOI:** 10.1186/s12903-025-07500-2

**Published:** 2025-12-18

**Authors:** Jiakang Zhu, Xianfeng Deng, Yake Wang, Cui Huang

**Affiliations:** State Key Laboratory of Oral & Maxillofacial Reconstruction and Regeneration, Key Laboratory of Oral Biomedicine Ministry of Education, Hubei Key Laboratory of Stomatology, School & Hospital of Stomatology, Wuhan University, Wuhan, 430079 China

**Keywords:** Transparent vinyl polysiloxane, Shore A hardness, Compressive modulus, Translucency, Light irradiance, Guided composite resin injection

## Abstract

**Background:**

Vinyl polysiloxane (VPS) index is the most commonly used tool of guided composite resin injection (G-CRI) technique. Understanding the mechanical and optical properties of various transparent VPS materials is vital for determining optimal G-CRI parameters and identifying the best material. Still, existing literature on this topic is limited. This study aimed to compare the mechanical and optical properties of various VPS materials used for G-CRI technique, and to evaluate the effect of material thickness on optical properties.

**Methods:**

Four transparent VPS materials (OKVD, DELIAN, HUGE, and ZHERMACK) were evaluated. Shore A hardness was measured on cylindrical specimens (Ø25 × 6 mm, *n* = 9) using a portable Shore durometer, and compression modulus was measured on specimens (Ø10 × 6 mm, *n* = 9) using a universal testing machine. For optical properties, cylindrical specimens (Ø12 mm, *n* = 5 per thickness) at five thickness (2, 4, 6, 8, and 10 mm) were fabricated. Translucency parameter was calculated from color measurements taken with a portable spectroradiometer against black and white backgrounds. Light irradiance transmitted through specimens was measured using a dental radiometer. Statistical analysis was performed using analysis of variance, and Pearson’s correlation was used to correlate the compressive modulus with Shore A hardness, and the light irradiance with translucency (α = 0.05).

**Results:**

The trend of changes in Shore A hardness and compressive modulus of VPS materials is consistent, with ZHERMARK > OKVD > DELIAN > HUGE. Both translucency and light irradiance decreased significantly with increasing thickness for all materials. DELIAN exhibited the highest optical properties across all thickness, while HUGE showed the lowest. Strong positive correlations were observed between Shore A hardness and compressive modulus (*r* = 0.93) and between translucency and light irradiance (*r* = 0.96–0.98).

**Conclusions:**

Significant variations exist in the mechanical and optical properties of transparent VPS materials, with ZHERMACK exhibiting superior mechanical properties and DELIAN superior optical properties. The optical properties declined significantly with increasing thickness. Transparent VPS material with higher mechanical properties can enhance the restoration accuracy of G-CRI technique, while thicker index or that fabricated using lower translucent materials necessitate longer exposure times for complete polymerization. Additionally, compressive modulus and light irradiance can be predicted using Shore A hardness and translucency, respectively.

## Background

Advancements in adhesive technology and composite resin materials have established direct composite resin restoration as a widely utilized treatment [1–3]. Because of its minimally invasive and inexpensive features, while still maintaining favorable esthetic outcomes, direct composite resin restoration has become the preferred option for most patient [4–6]. However, freehand direct composite resin restoration is time-consuming, technique-sensitive, and highly depend on the clinician’s experience and skill for optimal esthetic and functional results [7, 8]. Furthermore, achieving the designed contour in freehand direct composite resin restorations remains challenging, even for highly skilled dentists.

Guided composite resin injection (G-CRI) technique has been introduced in dentistry to address above limitations, enabling the rapid and accurate reproduction of pre-designed restoration contour [9–12]. G-CRI technique replicates the pre-designed contour of diagnostic wax-up through an index, followed by injection molding with highly-filled flowable composite resin to transfer the contour to the final restoration. Indices for G-CRI are primarily fabricated from two materials: transparent vinyl polysiloxane (VPS) or three-dimensional (3D) printing resin [9, 13–15]. Currently, the application of 3D printed index is constrained by its complex design process and the challenge of removal after restoration [13, 16]. In contrast, the VPS index is straightforward to fabricate and use, exhibits broad clinical applicability, and consequently remains the core tool of G-CRI technique [9, 15].

The elasticity of VPS index allows it to enter dental undercuts and facilitates easy removal after restoration [17]. However, this elasticity also carries a risk of deformation, compromising the restoration accuracy of G-CRI technique [15, 18]. Therefore, sufficient thickness is required for VPS index to provide the rigidity necessary to resist deformation. Given that the mechanical properties of VPS materials vary by brand, their index thickness requirements also differ [18, 19]. However, an increase in index thickness will reduce the transparency, thereby diminishing both surgical field visibility and the light irradiance required for restoration polymerization [18]. Clear visibility is essential for the G-CRI technique to prevent the formation of bubbles and overhangs in the restoration. Furthermore, light-curing parameters, notably the effective light irradiance and exposure time, significantly influence restoration quality [20–22]. Understanding the mechanical and optical properties of various VPS materials is therefore essential for determining appropriate index thickness and light-curing parameters for their application in G-CRI technique, and even identify the optimal material. Nevertheless, existing literature on this subject is notably limited.

Therefore, the purpose of the present study was to compare the mechanical (Shore A hardness and compression modulus) and optical (transparency and light irradiance) properties of different transparent VPS materials available in clinic, and to evaluate the effect of material thickness on these optical properties. The null hypotheses were that no significant difference would exist in the mechanical and optical properties among the tested transparent VPS materials, and that no significant difference would be observed in the optical properties of VPS materials at varying thicknesses.

## Methods

Four transparent VPS materials were tested in this study, including Transparency Regular (OKVD, Beijing, China), Delian Clear (DELIAN, Beijing, China), Transparent elastomeric impression material (HUGE, Shandong, China), and Elite Transparent (ZHERMACK, Badia Polesine, Italy). All tests were conducted under room temperature (23 ~ 25 °C).

### Shore A hardness

Cylindrical specimens (Ø25×6 mm) were fabricated using customized Teflon molds (*n*=9 for each material). The Teflon mold was placed on a glass plate covered with a mylar sheet. Transparent VPS material was injected into the mold and then covered with another mylar sheet and glass plate. A 10-kg weight was placed on the center of the glass plate to squeeze out excess material from the mold. After solidification, the final thickness of all specimens was verified by a digital caliper (DL91150; Deli, Zhejiang, China) and the error was controlled within ±0.1 mm.

A portable Shore durometer (LX-A, KOSLO, Guangdong, China) was used to measure Shore A hardness. The instrument was calibrated in compliance with the manufacturer’s instructions before measurement. The durometer was installed on a testing fixture, with its indenter positioned 10 mm away from the stage. Specimens were centered on the stage, and the actuation handle was depressed to ensure full indenter contact. Finally, hardness values were recorded within 1 s.

### Compressive modulus

Cylindrical specimens (Ø10 × 6 mm) were fabricated using customized Teflon molds as described above (*n* = 9 for each material). A universal testing machine (ElectroPlus E1000; Instron, MA, USA) was used to measure compressive modulus. Specimen was centered between two testing fixtures with opposing metal platens. The upper platen was actuated downward at a constant crosshead speed of 10 mm/min under displacement control, achieving 2 mm of axial compression that induced 33.3% strain in the specimen. Load data were acquired at 20 ms intervals. Compressive modulus was calculated using the internal software of the universal testing machine (Bluehill 2; Instron, MA, USA).

### Translucency

Cylindrical specimens (Ø12 mm) with five thickness (2, 4, 6, 8, and 10 mm) were fabricated using customized Teflon molds (*n* = 5 for each material and thickness), according to the method described above. A portable spectroradiometer (DS-410; CHN Spec, Zhejiang, China) was used for the color measurements for all specimens against both black and white backgrounds. The spectrophotometer was calibrated in compliance with the manufacturer’s instructions before each measurement. The spectroradiometer’s probe (Ø5 mm) faced the center of the specimen and maintained intimate contact without coupling medium during measurement. L^*^, a^*^, and b^*^ color coordinates based on the CIELAB system were recorded. Three short-term repeated measurements were performed for each specimen, and the results were averaged to obtain a single value for the specimen.

The relative translucency parameter (RTP) value for each specimen was calculated using the color coordinate values between the readings over the black and white backgrounds, according to the following formula [23]:

$$\begin{aligned} &{\mathrm{RTP}}_{CIEDE2000}\\&=\sqrt{{\left(\frac{{L}_{B}{\prime}-{L}_{W}{\prime}}{{K}_{L}{S}_{L}}\right)}^{2}+ {\left(\frac{{C}_{B}{\prime}-{C}_{W}{\prime}}{{K}_{C}{S}_{C}}\right)}^{2}}\\&+{\left(\frac{{H}_{B}{\prime}-{H}_{W}{\prime}}{{K}_{H}{S}_{H}}\right)}^{2}+{R}_{T}\left(\frac{{C}_{B}{\prime}-{C}_{W}{\prime}}{{K}_{C}{S}_{C}}\right)\left(\frac{{H}_{B}{\prime}-{H}_{W}{\prime}}{{K}_{H}{S}_{H}}\right) \end{aligned}$$

where the subscripts “B” and “W” referred to the lightness (L′), chroma (C′), and hue (H′) of the specimens against black and white backgrounds, respectively.

### Light irradiance

The same specimens used for translucency testing were subsequently employed to measure light irradiance using a dental radiometer (Bluephase Meter II; Ivoclar Vivadent, Schaan, Liechtenstein). The working mode of the radiometer was selected corresponding to the tip diameter (10 mm) of the light-curing unit (LCU) (Bluephase N; Ivoclar Vivadent, Schaan, Liechtenstein) used in this study. Prior to each VPS material measurement, baseline light intensity (mW/cm^2^) was measured five times by recording the LCU output directly at the center of the radiometer’s measuring groove surface (0 mm). Subsequently, each specimen was centered within the measuring groove with the LCU tip firmly contacting its surface. Specimen was light exposed using LCU in “high” mode for 5 s, and transmitted light irradiance (mW/cm^2^) was recorded. Additionally, irradiance measurements were taken five times at 0, 2, 4, 6, 8 and 10 mm away from the measuring groove surface without the interposition of the PVS material, which was used as a control group.

Based on measured light irradiance values, exposure time required to achieve specific radiant exposure thresholds (J/cm^2^) was calculated for each VPS material and no VPS material across varying thicknesses/distances. This calculation adheres to the exposure reciprocity law governing light-polymerization of composite resin.

### Statistical analysis

The sample size was determined a priori using G*Power software (version 3.1.9.6 for Mac; Heinrich Heine University Düsseldorf, Germany). For the mechanical properties test, which involved 4 material-based subgroups, a total of 36 specimens (9 per group) was calculated to achieve a power > 0.8, with an effect size of 0.6 and α = 0.05. For the optical properties test, which included 20 subgroups based on material and thickness, a total of 82 specimens was required to achieve the same power and significance level. Therefore, the sample size for each group was 5.

The data were presented as the mean and standard deviation values. The result of the Shapiro–Wilk test showed that the data presented normal distribution (*p* > 0.05). One-way analysis of variance (ANOVA) with the least significant difference (LSD) test was used to assess the effect of material type on Shore A hardness and compressive modulus. Two-way ANOVA with the Bonferroni correction was used to assess the material type, thickness, and their interaction on RTP and light irradiance. Pearson’s correlation was used to correlate the compressive modulus with Shore A hardness, and the light irradiance with translucency. The test standard was bilateral (α = 0.05 for all tests). Statistical analyses were performed with SPSS software (version 26.0; SPSS, Chicago, USA).

## Results

**Fig. 1:**
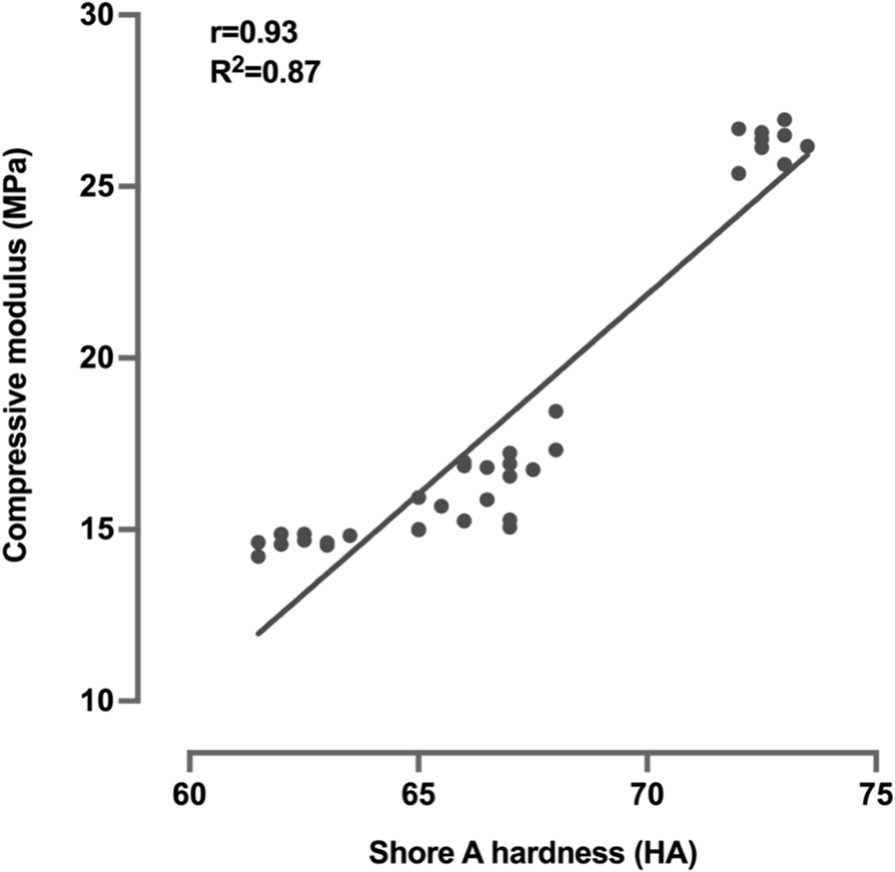
Linear correlation between compressive modulus and Shore A hardness of transparent VPS materials

The Shore A hardness and compressive modulus of all transparent VPS materials were showed in Table 1, both exhibiting a trend of ZHENMARK > OKVD > DELIAN > HUGE. One-way ANOVA revealed that material type had a significant effect on both Shore A hardness (df = 3, F = 332.258, *p* < 0.001) and compressive modulus (df = 3, F = 1409.788, *p* < 0.001), with statistically significant differences in all pairwise comparisons among the four materials. Pearson’s correlation analysis revealed that there was a strong positive linear correlation between Shore A hardness and compressive modulus, with *r* = 0.93 and *R*^2^ = 0.87 (Fig. 1).

**Table 1:** Shore A hardness and compressive modulus of all transparent VPS materials

Material	Shore A hardness (HA)	Compressive modulus (MPa)
OKVD	67.0 ± 0.75^C^	17.1 ± 0.56^C^
DELIAN	65.9 ± 0.82^B^	15.4 ± 0.37^B^
HUGE	62.4 ± 0.70^A^	14.6 ± 0.21^A^
ZHERMACK	72.7 ± 0.50^D^	26.3 ± 0.50^D^

Same superscript uppercase letter indicates no significant difference between different groups (*p* > 0.05)

**Fig. 2:**
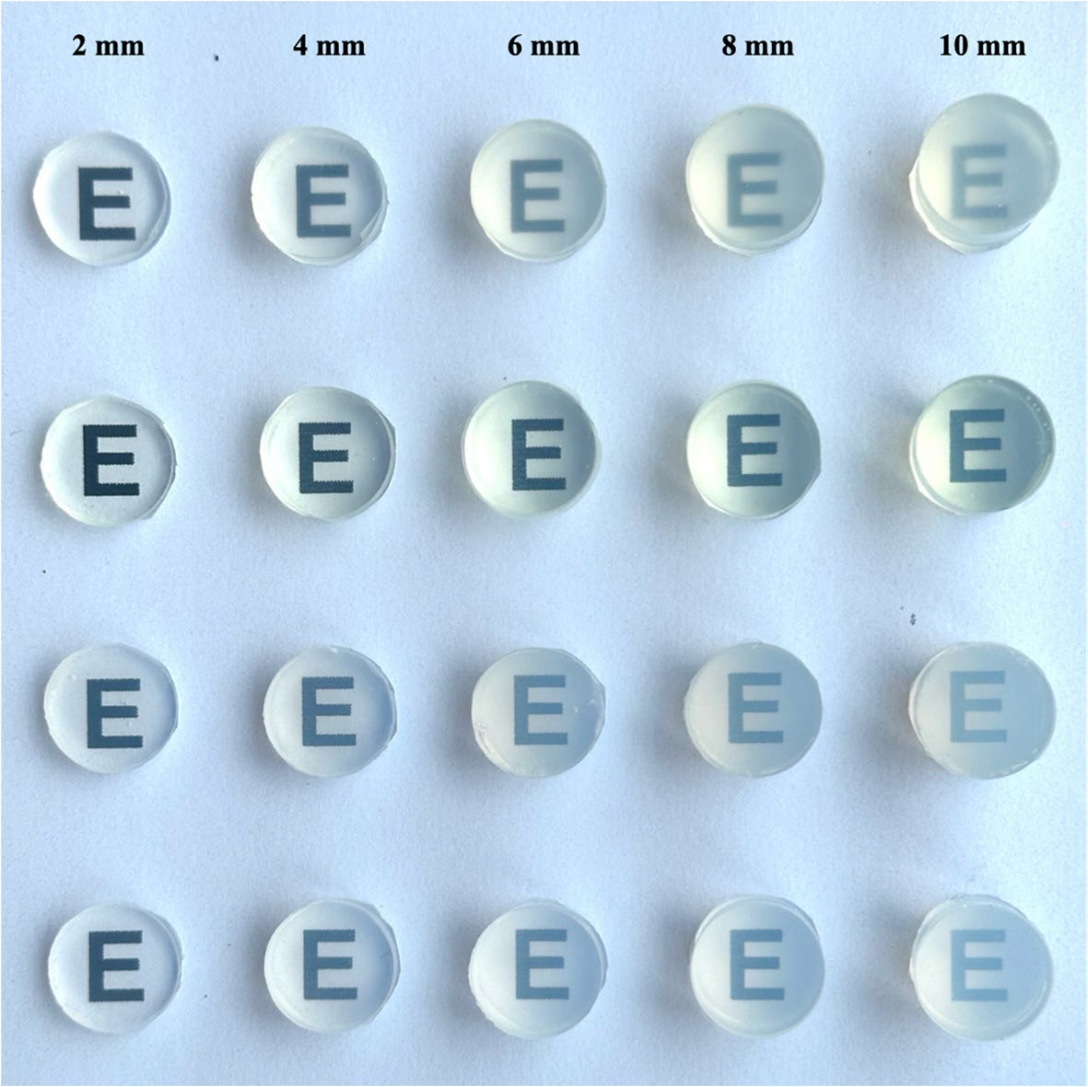
Representative specimens displaying translucency for each thickness of four transparent VPS materials. The first to fourth rows are OKVD, DELIAN, HUGE, and ZHERMACK

The representative specimens of the four transparent VPS materials at each thickness were showed in Fig. 2. The letter “E” was clearly readable across all materials and thicknesses, with DELIAN exhibiting superior clarity. Two-way ANOVA revealed that material type, thickness, and their interaction had significant effects on both RTP and light irradiance (Table 2).

**Table 2:** Two-way ANOVA results of RTP and light irradiance

Objective	Source	df	F	*p*
RTP	Thickness	4	2669.124	< 0.001
	Material type	3	114.060	< 0.001
	Thickness × Material type	12	4.110	< 0.001
Light irradiance	Thickness	4	7910.094	< 0.001
	Material type	3	770.677	< 0.001
	Thickness × Material type	12	28.336	< 0.001

**Fig. 3:**
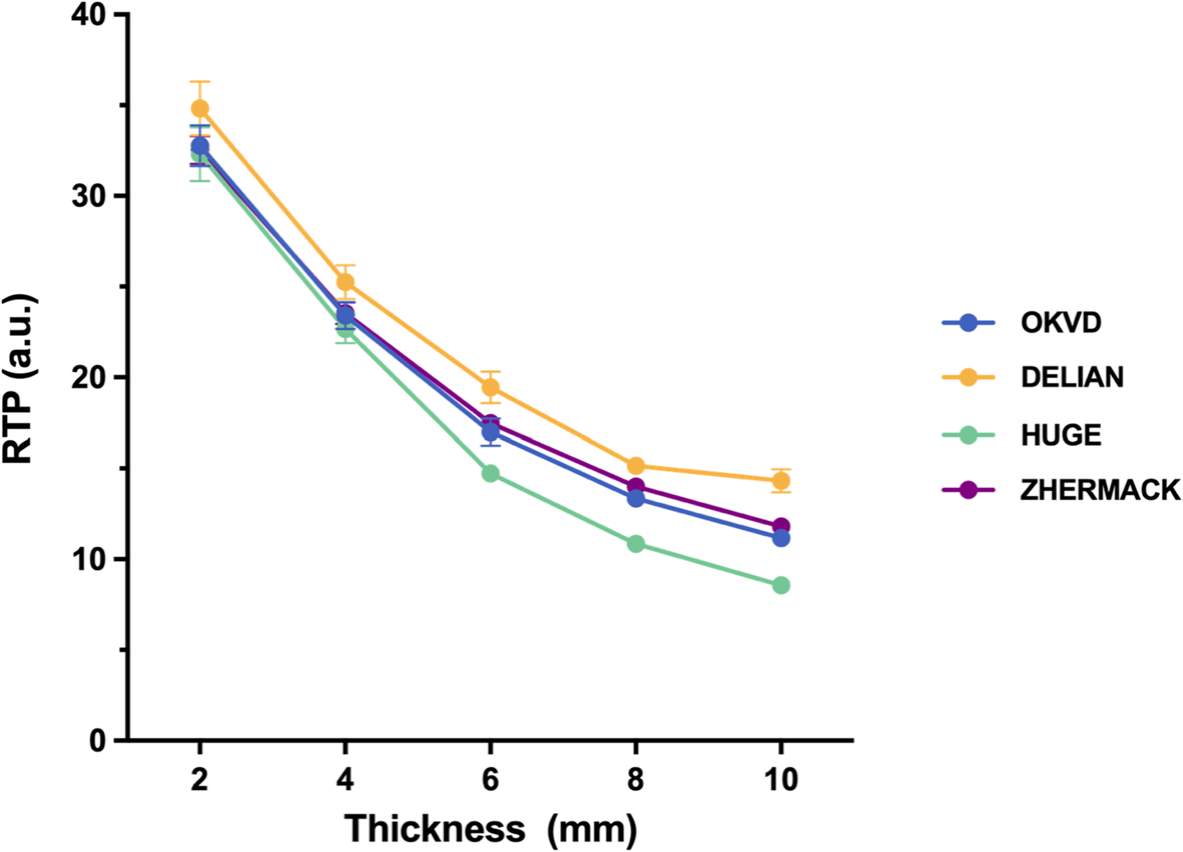
RTP for each thickness of four transparent VPS materials

Figure 3 showed the RTP values and trends of the four VPS materials across different thicknesses. The RTP values decreased significantly with increasing thickness for all materials. Notably, DELIAN consistently exhibited the highest RTP values across all thicknesses, while HUGE maintained the lowest. No significant differences were found between OKVD and ZHERMACK at any thickness (all *p* = 1.000). Besides, there were no significant differences for HUGE vs. OKVD or HUGE vs, ZHERMACK at 2 mm (both *p* = 1.000) and 4 mm (*p* = 0.756 and 0.447, respectively). At 8 mm, no significant differences were found between DELIAN and ZHERMACK (*p* = 0.121).

**Fig. 4:**
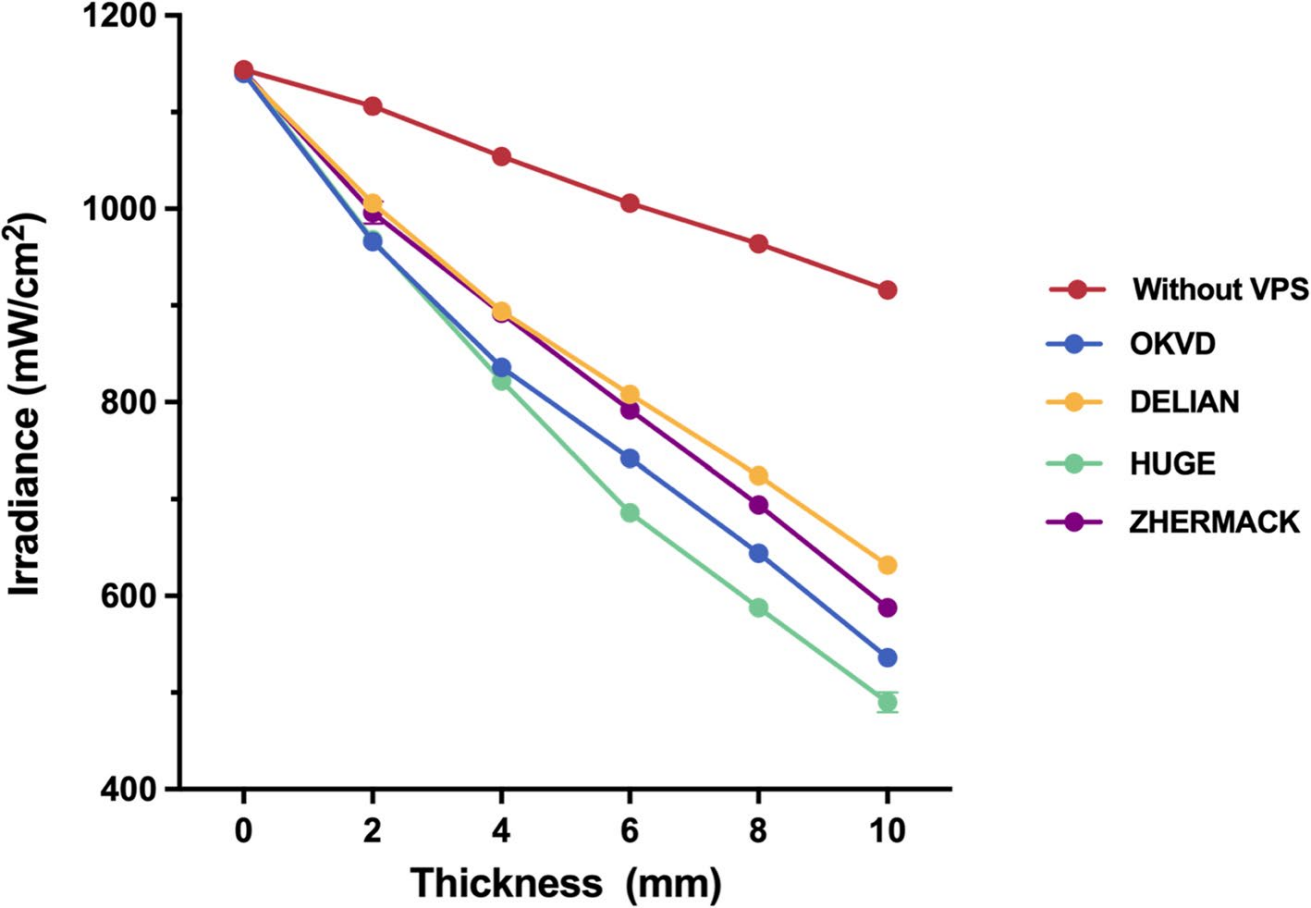
Light irradiance for each thickness of four transparent VPS materials and the control group (no VPS)

**Fig. 5:**
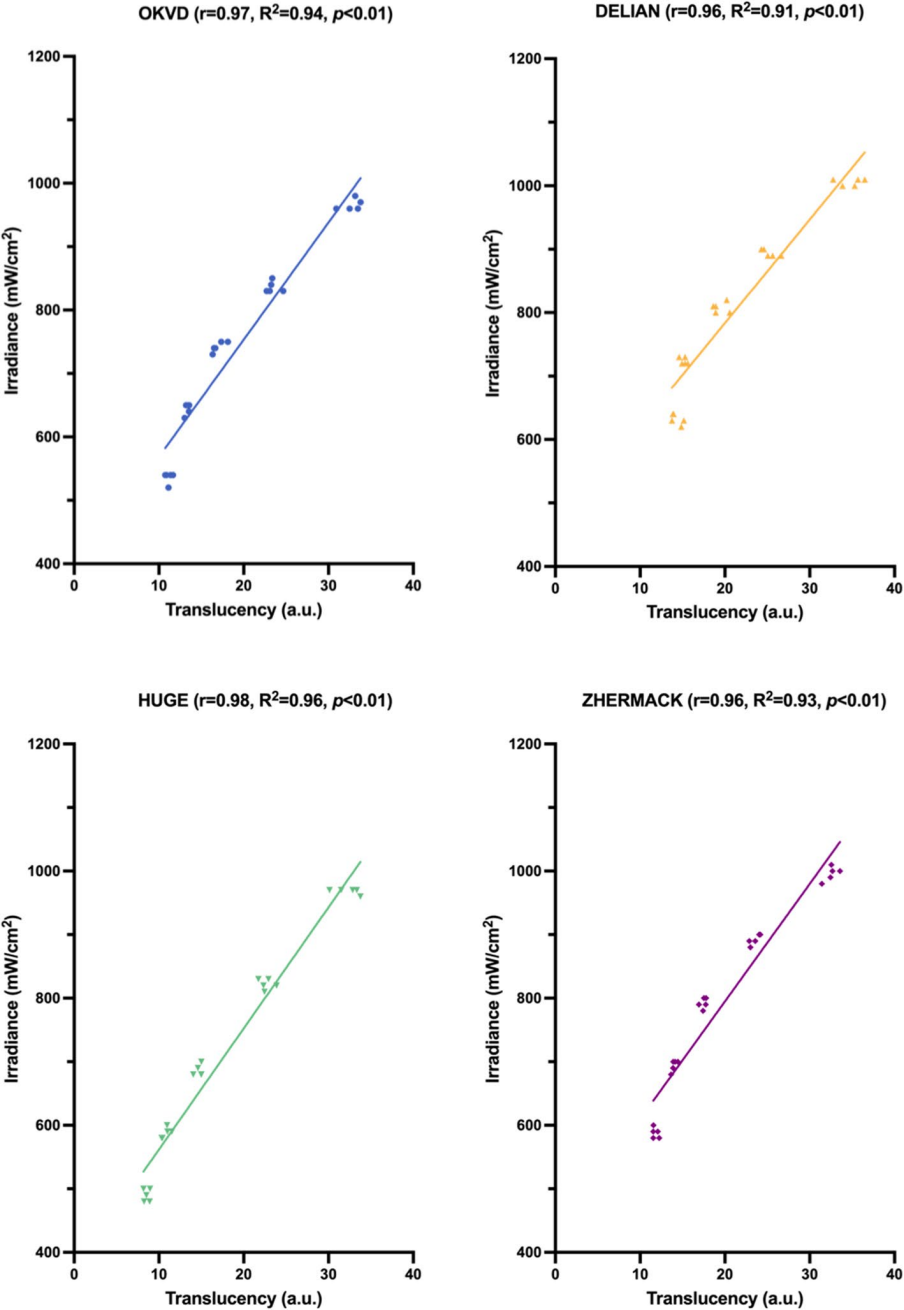
Linear correlation between light irradiance and RTP of four transparent VPS materials with thickness variation

Figure 4 showed the variation of light irradiance of the LCU used in this study across different distances, as well as the trends of light irradiance across different materials and thicknesses. The light irradiance of LCU itself was 1144.0 ± 5.5 mW/cm^2^ at 0 mm, gradually decreasing to 916.0 ± 5.5 mW/cm^2^ at 10 mm. The light irradiance decreased significantly with increasing thickness for all VPS materials and was significantly lower than the control group (no VPS) at equivalent distances. DELIAN exhibited the highest light irradiance across all thicknesses, with no significant differences versus ZHERMACK at 2 mm (*p* = 0.362) and 4 mm (*p* = 1.000). HUGE exhibited the lowest light irradiance across all thicknesses, with no significant differences versus OKVD at 2 mm (*p* = 1.000) and 4 mm (*p* = 0.056). A strong positive linear correlation existed between RTP and light irradiance, with r ranging from 0.96 to 0.98, and R^2^ ranging from 0.91 to 0.96 (Fig. 5).

**Fig. 6:**
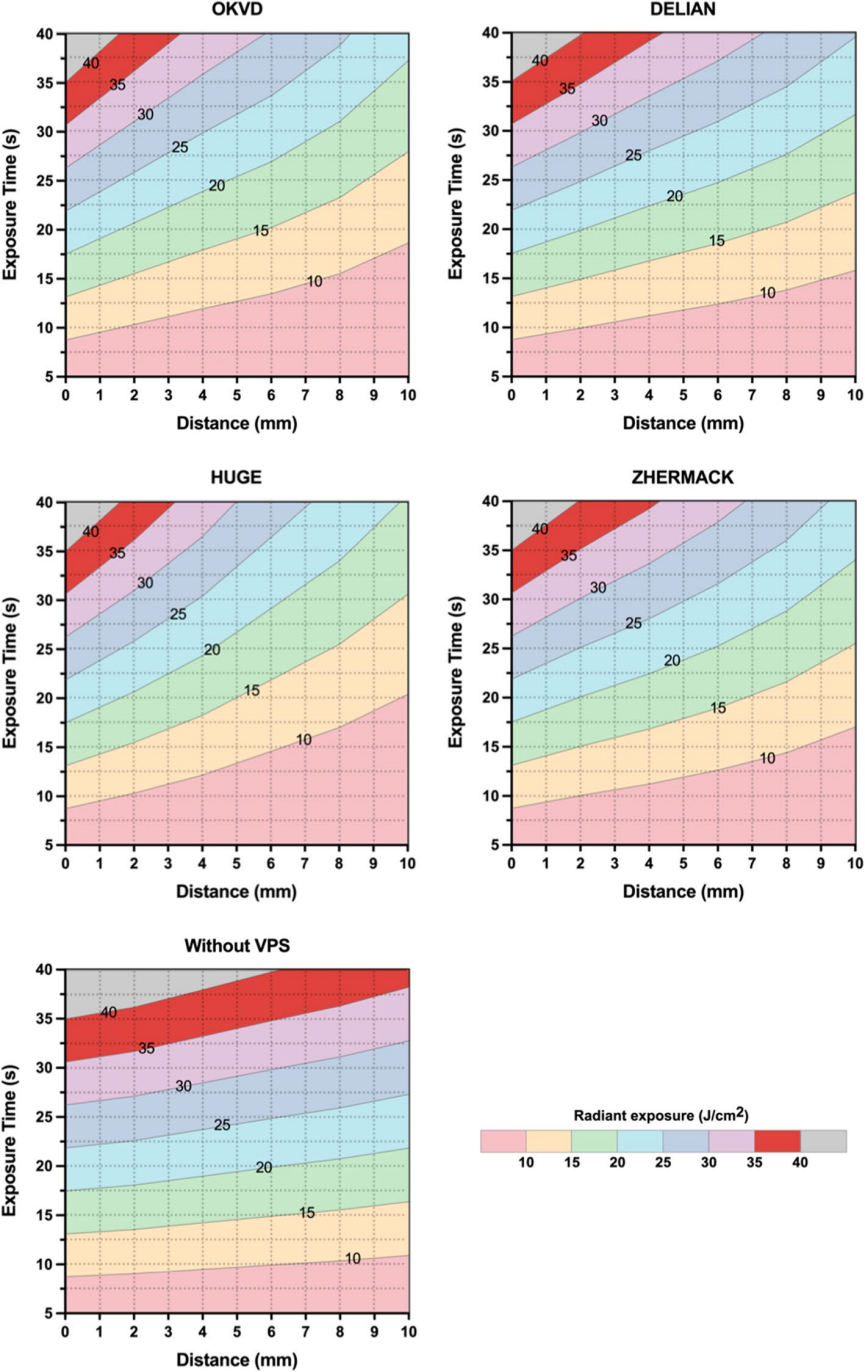
Exposure time–distance relationship across different radiant exposure thresholds for different transparent VPS materials and control (no VPS)

Figure 6 demonstrated the correlation between exposure time and working distance required to attain specific radiant exposure thresholds across various transparent VPS materials and the control group. In the control group (no VPS), achieving a target radiant exposure of 15 J/cm^2^ required approximately 12 s of exposure at 1 mm distance, increasing to 16 s at 10 mm distance. Material type and thickness significantly influenced exposure time in VPS groups: DELIAN required 15 s at 2 mm thickness but demonstrated a 60% increase (24 s) when thickness reached 10 mm. Similarly, HUGE required 16 s at 2 mm thickness while exceeding 30 s at 10 mm thickness for equivalent radiant exposure.

## Discussion

The results indicated that there were significant differences in all mechanical (including Shore A hardness and compressive modulus) and optical (including translucency and light irradiance) properties among the transparent VPS materials. Furthermore, significant statistical differences were found in the optical properties of transparent VPS materials at varying thicknesses. Therefore, the null hypotheses of this study were rejected.

Variations in the mechanical properties across different brands of transparent VPS materials correlate with material composition. Previous studies have shown that as the molecular weight of the base silicone molecule decreases or the content of crosslinking agents and reinforcing fillers increases, the mechanical properties of VPS materials show an enhanced trend [24, 25]. Utilizing transparent VPS material with a higher compressive modulus for injection indices fabrication can improve the restoration accuracy of G-CRI technique. Throughout the restoration process, the index must be manually stabilized with finger pressure to ensure proper fit [14]. Thus, the VPS index requires sufficient thickness to prevent deformation during manipulation.

The mechanical properties of the material, such as shore A hardness and compressive modulus, are inherent and do not change with specimen thickness. However, as thickness increases, the index exhibits greater macroscopic stiffness. In other words, for a given thickness, a material with a higher compressive modulus will produce a stiffer structure. Zhu et al. reported that a minimum thickness of 4 mm is necessary when using Elite Transparent (ZHERMACK) for index fabrication in G-CRI technique to achieve satisfactory clinical outcomes [15]. The present results identified ZHERMACK as exhibiting the optimal mechanical properties among the tested materials, with a Shore A hardness of 72.7 HA and a compressive modulus of 26.3 MPa. Therefore, when other VPS materials involved in this study are used for G-CRI technique, a thickness exceeding 4 mm is required, but the specific minimum thickness necessitates further investigation. Furthermore, the structural stiffness of the index is influenced by its span length and the number of supporting teeth, which in turn is critical for restoration accuracy. Notably, longer unsupported spans are more prone to flexural deformation when using an equivalent thickness. Therefore, for multi-unit or full-arch applications, using materials with superior mechanical properties or increasing the index thickness are recommended. Alternatively, a segmented injection approach should be considered. However, increased index thickness reduces transparency, compromising internal visibility and potentially affecting the quality of injected composite resin restorations. In this study, as material thickness increased, all VPS materials exhibited the greatest reduction in translucency between 2 and 4 mm, ranging from 28 to 35%. Meanwhile, the minimum reduction occurred between 8 and 10 mm, with HUGE showing a 21% decrease, OKVD and ZHERMACK at 16%, and DELIAN as low as 5%. During the injection of flowable composite resin, care must be taken to prevent the formation of bubbles, which can cause defects and failures in restorations. Simultaneously, attention should be paid to the amount of injected composite resin and the overflow location of excessive materials to avoid excessive pressure within the index. Excessive internal pressure can cause separation between the index and teeth, forcing excess material into the resulting gap and creating overhangs that complicate subsequent treatment procedures.

Light irradiance decreased for all materials with increasing thickness. Nevertheless, adequate radiant exposure for complete composite resin polymerization can be achieved by extending light-curing time, thereby ensuring good mechanical performance and adhesive strength of the restoration [22, 26]. The radiant exposure heat maps in Fig. 6 provide a theoretical basis for the selection of light-curing parameters when using various VPS materials and thicknesses in the G-CRI technique. Significantly, the results of this study were obtained under idealized conditions. Clinically, the convex surface of index prevents full contact between the index and the curing light tip, which will further lead to attenuation of light irradiance [14, 15]. Clinicians should therefore extend exposure time or perform additional light-curing after index removal to ensure complete polymerization.

Measuring the compressive modulus of VPS materials requires complex testing conditions and expensive equipment. In contrast, Shore A hardness measurement employs simple conditions and inexpensive, portable equipment [27]. Pearson’s correlation analysis revealed that there was a strong positive linear correlation between Shore A hardness and compressive modulus. Therefore, Shore A hardness enables estimation and predication of the compressive modulus, facilitating index thickness determination in the G-CRI technique. Similarly, a strong positive linear correlation was observed between translucency and light irradiance, which supports using the translucency of VPS material to guide exposure time determination.

This study serves as a valuable reference for selecting VPS materials in the G-CRI technique by evaluating their mechanical and optical properties. For clinical decision-making, however, dentists must also integrate other practical factors (such as working time, cost, and reusability) to ultimately identify the most suitable material for their specific clinical conditions. A limitation of this study is its idealized and standardized in vitro design. Specimens were fabricated against perfectly smooth glass plates and Mylar sheets, resulting in flawlessly flat and smooth PVS surfaces. Clinically, VPS indices were fabricated against stock impression trays, thermoformed acetate plate trays, or 3D printed trays, which can create a slightly rough surface for the index and reduce translucency [14, 15, 18]. Bedsides, the mechanical properties of VPS materials may be lower under intraoral conditions due to their temperature-dependent viscoelastic behavior. Furthermore, variations in LCU tip shape, size, and light intensity mean the attenuation trend of light irradiance through VPS materials may differ from our observations [28, 29]. Therefore, further studies are needed to investigate the effects of the VPS index’s convex geometry, surface roughness, and LCU type on its optical properties.

## Conclusions

Within the limitations of the present study, it was concluded that:

1. Shore A hardness and compression modulus vary across VPS materials. Elite Transparent exhibits superior mechanical properties among tested VPS materials, indicating its potential to improve the restoration accuracy of G-CRI technique.
2. Translucency and transmitted light irradiance vary across VPS materials and decrease with increasing material thickness. Transparency Regular exhibits optimal optical properties, indicating its ability to provide better visibility and light-curing condition for G-CRI technique.
3. Shore A hardness and translucency show strong positive correlations with compression modulus and light irradiance, respectively, suggesting that compressive modulus and light irradiance of VPS materials can be evaluated and predicted by Shore A hardness and translucency.

## Data Availability

The data of this study are available from corresponding author on reasonable request.

## Abbreviations

VPS: Vinyl polysiloxane
G-CRI: Guided composite resin injection
RTP: Relative translucency parameter
LCU: Light-curing unit
ANOVA: Analysis of variance
LSD: Least significant difference
